# ANCA positive crescentic glomerulonephritis outcome in a Central East European cohort: a retrospective study

**DOI:** 10.1186/s12882-015-0091-8

**Published:** 2015-06-30

**Authors:** Iuliana Andreiana, Simona Stancu, Andreea Avram, Ludmila Taran, Gabriel Mircescu

**Affiliations:** Department of Nephrology and Dialysis, “Dr. Carol Davila” Teaching Hospital of Nephrology, 4th Calea Grivitei Street, Bucharest, 010731 Romania; Department of Internal Medicine and Nephrology, “Carol Davila” University of Medicine and Pharmacy, Bucharest, Romania

**Keywords:** ANCA, Outcome, Pauci-immune glomerulonephritis, Histopathological classes

## Abstract

**Background:**

The recently suggested distinct pathogenic pathways for myeloperoxidase (MPO) and proteinase 3 (PR3) anti-neutrophilic cytoplasmic antibodies (ANCA) associated vasculitis could result in different modes of presentation and outcome. Moreover, kidney outcome was related to a new histopathologic classification of pauci-immune glomerulonephritis. As reports were not always concordant, possible because differences in severity of organ lesions and ethnicity, we evaluated the outcome of a cohort of Central-East European patients with crescentic glomerulonephritis in relation with ANCA specificity and histopathological classification.

**Methods:**

Seventy-five patients were consecutively diagnosed by kidney biopsy (76 % MPO-ANCA specificity, 52 % crescentic) and followed for a median period of 3.2 years. Study end-points were response to therapy, end stage renal disease (ESRD) and death.

**Results:**

PR3-ANCA patients were younger, in higher proportion male and had higher Birmingham Vasculitis Activity Scores (BVAS). The kidney disease was severe at presentation (median creatinine 5 mg/dL; 27 % required temporary dialysis) and worst in PR3-ANCA positive patients (50 % patients needed temporary dialysis vs. 19 %). The lung was the second most affected organ (31 % severe lung hemorrhage). Lung and kidney damage were related; the odds of hemorrhagic alveolitis in patients needing dialysis at presentation were 4 (95 % CI 1–13; *p* = 0.006) times higher than in those who did not. The rate of response to therapy (without signs of active vasculitis and stable or declining serum creatinine) was 60 % and was associated with dialysis independency, older age and higher platelet number at presentation. The probabilities to survival 1 and 5 years for kidney and patient were 93 and 64 %, and respectively 88 and 67 %. Kidney survival was predicted by response to therapy and dialysis independency at presentation. Patients with BVAS < 15 and responding to induction therapy had better chances of survival. Neither response to therapy nor outcome was influenced by ANCA specificity or by the histopathological class.

**Conclusions:**

When kidney damage is severe in ANCA vasculitis, the need of dialysis at presentation and the response to induction therapy overcome the prognostic utility of both ANCA specificity and histopathological class.

## Background

ANCA-associated vasculitis is a rare, but severe autoimmune disease [1–3]. Kidney involvement (i.e. pauci-immune, crescentic glomerulonephritis), is frequent in ANCA vasculitis [4, 5] and can cause acute renal injury, which is potentially fatal, mostly when associated with pulmonary hemorrhage [6, 7]. Accordingly, patients seen in a nephrology setting may have distinct clinical features from those seen by rheumatologists or pneumologists and they raise complex therapeutic problems. The relatively few reported series which addressed patients with severe kidney disease suggested a poorer outcome in this set of patients [1, 2, 8, 9]. Whether ANCA type is related to the severity of vasculitis in patients seen by nephrologists is a matter of debate [1, 2, 4]. Recent data suggest distinct pathogenic pathways for myeloperoxidase (MPO) and proteinase 3 (PR3) ANCA vasculitis, which could result in different modes of presentation and outcome, although overlapping features are not infrequent [10]. However, reports from different groups are not always concordant, possibly because of differences in ethnicity (PR3-ANCA is rare in Asians and in African Americans, where MPO-ANCA is frequent) and in geographical distribution (in Europe there is a North to South gradient of incidence in PR3 and a South to North gradient in MPO) [11–14]. Thus, the potential differences in phenotype between MPO and PR3-ANCA are still to be defined [10].

The histological features of kidney biopsy could have a certain relationship to the outcome. Older studies have suggested that the proportion of normal glomeruli was a predictor of a good renal outcome [15], while the inflammatory lesions [16], i.e. the proportion of crescents, predicted the response to immunosuppressive therapy and the proportion of sclerotic glomeruli was related to a worse renal prognosis [17, 18]. More recently, a panel of international renal pathologists proposed and validated the first classification for ANCA vasculitis: patients in the sclerotic class (≥50 % sclerotic glomeruli) had the worst renal prognosis, followed in order by those in the mixed (<50 % normal, <50 % crescents, <50 % sclerotic), crescentic (≥50 % crescents) and focal (≤50 % crescents) class [19]. The prognostic utility of this classification was confirmed in some but not all subsequent studies [20–23].

We aimed to evaluate ANCA phenotypes, kidney and patient outcome in a cohort of Central-East European patients with severe crescentic glomerulonephritis admitted in a tertiary Nephrology center in relation with ANCA specificity and histopathological classification.

## Methods

### Patients

This is a retrospective cohort study on all adult patients consecutively diagnosed by kidney biopsy with pauci-immune crescentic glomerulonephritis and followed in a single Nephrology center serving a population of about 4.85 million. From January 2000 till January 2014, pauci-immune crescentic glomerulonephritis were diagnosed in 104 patients. Thirteen patients were excluded because they were ANCA negative, 4 because of incomplete data and 12 were lost to follow-up.

The final cohort comprised 75 patients with ANCA positive pauci-immune crescentic glomerulonephritis who were followed over a median period of 3.2 [0.1; 5.5] years.

All subjects signed an informed consent form authorising us to use their demographic and medical data in this study.

The study was performed in accordance with Helsinky Declaration and it was approved by the local Ethics Committee of the “Dr. Carol Davila” Teaching Hospital of Nephrology.

### Diagnosis and follow-up

The criteria for pauci-immune crescentic glomerulonephritis diagnosis were crescents (cellular and/or fibrous) in at least 50 % of examined glomeruli by light microscopy and a direct immunofluorescence assay of 0 to 1+ on a scale from 0 to 4+ [24]. For this study, all the biopsies were reviewed by our pathologist and they were classified in four classes as proposed by Berden et al.: crescentic (≥50 % glomeruli with cellular crescents), focal (≥50 % normal glomeruli), sclerotic (≥50 % sclerotic glomeruli) or mixed (<50 % normal glomeruli, <50 % glomeruli with cellular crescents, <50 % sclerotic glomeruli ) [19].

ANCAs were assessed by capture PR3-ANCA and MPO –ANCA ELISA (Euroimmun™, Lübeck, Germany) or by indirect immunofluorescence (monoclonal mouse anti-human myeloperoxidase antibodies Dako™, Glostrup, Denmark).

Markers of kidney damage were proteinuria measured in 24 h urine collection, hematuria (macroscopic and microscopic, red blood cells casts), serum creatinine and the histopathologic classes.

Hemorrhagic alveolitis was diagnosed based on chest radiographs and acute anemia without evidence of another external bleeding, and was classified as severe when oxygen partial pressure in arterial blood decreased under 60 mmHg. Severe anemia was defined as hemoglobin level less than 7 g/dL.

Inflammation was assessed using erythrocyte sedimentation rate (ESR), serum fibrinogen, serum albumin, platelet count and white blood cells count.

The measurements were performed by standard laboratory methods on an Olympus AU 400 chemistry auto-analyzer, and a MINDRAY BC 3000 hematology auto-analyzer.

The severity of vasculitis was evaluated using Birmingham Vasculitis Activity Score ver. 3 (BVAS) [25].

The follow-up protocol included monthly visits with clinical (BVAS) and laboratory evaluation, until remission and every 3 months thereafter.

Response to therapy was defined as disappearance of hematuria, stable or improving serum creatinine and no signs of activity in other organs [26].

Dialysis therapy was considered temporary when needed for less than 3 months.

Study end-points were response to therapy, end stage renal disease (ESRD) and death.

### Therapy

Induction therapy was done with steroids (3 daily intravenous pulses with methylprednisolone 0.5–1 g then prednisone 0.5 mg/kg daily, gradually tapered after one month) associated with cyclophosphamide 0.5 g/m^2^, two pulses in the first month, then one pulse monthly for the next five months in the majority of patients (the mean cumulative cyclophosphamide dose was 4.6 g). Maintenance therapy consisted in prednisone (10 mg daily in the first year and 7.5 mg daily afterwards) and azathioprine 1.5–2 mg/kg per day for at least 2 years. Plasmapheresis was only occasionally performed (5 patients), usually in case of severe hemorrhagic alveolitis, because this therapy is not reimbursed by the health insurance authority.

### Statistical analysis

Categorical variables are presented as percentages and comparison tests were performed using Pearson χ2 test. Continuous variables are displayed as mean with 95 % confidence interval (95 % CI) or median and quartiles [1; 3], according to their distribution. Comparisons were done with ANOVA, Mann-Whitney and Kruskal-Wallis tests, as appropriate.

Survival analyses were conducted with the Kaplan-Meier method and Mantel-Cox log-rank test for comparisons. Models of binomial or multinomial logistic regression were built to estimate differences between groups at presentation. Multivariate Cox proportional hazard ratios (PHR) models were used to evaluate parameters associated with various outcomes.

A *p* ≤ 0.05 was considered statistically significant.

Statistical analyses were performed with SPSS (SPSS Inc., Chicago, IL) and Analyse-it™ (Analyse-it Software, Ltd., Leeds, UK) packages.

## Results

### Patients’ characteristics at presentation

Table 1 shows the baseline characteristics of patients. The clinical condition at presentation was severe (median BVAS 17). All patients had severe kidney disease (median creatinine 5 mg/dL, 27 % needed dialysis). The lung was the second most affected organ; 31 % of patients had severe hemorrhagic alveolitis. The odds of hemorrhagic alveolitis in patients needing dialysis at presentation were 4 (95 % CI 1–13; *p* = 0.006) times higher than in those who did not.

**Table 1:** Patients’ characteristics at presentation

Parameter	All *n* = 75	PR3-ANCA *n* = 18	MPO-ANCA *n* = 57	*P* value
General				
Gender (% Male)	48	78	39	0.004
Age >65 years (%)	35	11	42	0.02
Age (years)	60 [53; 68]	58.0 [43; 62]	63 [55; 69]	0.01
Cold season at diagnosis	60 %	65 %	62 %	0.81
Time to diagnosis (mo)	2.0 [1.0; 5.6]	1.5 [1.0; 3.0]	2.0 [1.0; 6.0]	0.12
Vasculitis symptoms				
Constitutional (%)	84	94	81	0.17
Skin (%)	9	17	7	0.22
Ocular (%)	3	11	0	0.01
Upper respiratory (%)	8	22	4	0.01
Lung (%)	48	50	47	0.85
Severe lung hemorrhage	31 %	39 %	28 %	0.39
Kidney (%)	100	100	100	1.00
Digestive (%)	4	0	5	0.32
CNS (%)	4	0	5	0.32
Organs affected (No)	2 [1; 2]	2 [1; 3]	2 [1; 2]	0.08
BVAS	17 [15; 21]	21 [15; 22]	17 [15; 20]	0.05
Inflammation				
Hemoglobin (g/dL)	8.5 [7.5; 9.8]	7.7 [5.7; 9.5]	9.0 [7.8; 9.8]	0.04
Severe anemia (%)	35	56	28	0.03
WBC (per mm^3^)	9750 [7800; 14400]	10700 [7800; 15075]	9650 [7683; 13900]	0.64
ESR (mm/1 h)	97 [64; 120]	100 [80; 140]	94 [63; 120]	0.18
Fibrinogen (mg/dL)	680 [568; 800]	714 [608; 806]	670 [547; 800]	0.48
Serum albumin (mg/dL)	3.6 [3.10; 4.0]	3.3 [2.9; 3.7]	3.7 [3.1; 4.0]	0.15
Kidney disease				
Histopathologic class				
Crescentic (%)	52	67	47	0.52
Focal (%)	5	6	5	
Mixed (%)	32	22	35	
Sclerotic (%)	11	6	12	
Creatinine (mg/dL)	5.0 [3.4; 7.9]	7.2 [4.2; 10.0]	4.8 [3.1; 7.2]	0.04
Proteinuria (g/day)	0.8 [0.5; 2.2]	1.0 [0.4; 1.4]	0.8 [0.5; 2.3]	0.73
Hematuria (per mm^3^)	260 [190; 800]	535 [288; 1200]	230 [183; 623]	0.005
Macroscopic hematuria	44 %	78 %	33 %	0.001
RBC casts (%)	37	72	28	0.02
Dialysis at presentation	27 %	50 %	19 %	0.01

Values are median [quartile 1; quartile 3] if not otherwise specified

*mo* months, *CNS* Central nervous system, *No* number, *BVAS* Birmingham Vasculitis Activity Score, *WBC* white blood cells, *ESR* erythrocyte sedimentation rate, *RBC* red blood cells

**Fig. 1:**
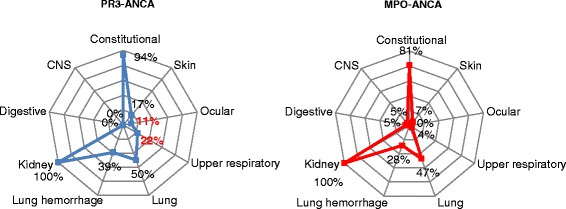
Prevalence of organs affected according to ANCA serology. Legend: numbers represent percent from 75 patients which presented with the mentioned type of vasculitis manifestation; percents in red means statistically significant difference (*p* < 0.05). CNS Central nervous system

Most patients (76 %) were MPO-ANCA positive. PR3-ANCA patients were younger, in higher proportion male, had higher BVAS, resulting from more frequent upper respiratory tract and eye symptoms and more severe kidney damage (Table 1, Fig. 1).

Most of the patients (52 %) were in the crescentic histopathologic class. PR3-ANCA and MPO-ANCA specificities had a similar distribution in the histopathologic classes (Table 1).

When evaluated by logistic regression, in patients with creatinine ≥5 mg/dL, PR3-ANCA was associated with male gender and dialysis at presentation (Table 2).

**Table 2:** Differences in clinical presentation according to ANCA serology

Variables in equation^a^	B	S.E.	Exp(B)	95 % CI		Sig.^b^
Gender (Male)	2.92	1.20	18.61	1.76	196.91	0.02
Dialysis at presentation (Yes)	2.10	0.92	8.16	1.34	49.56	0.02
Constant	−0.93	0.68	0.40			0.17

^a^Selection variable: Creatinine ≥5 mg/dL

^b^Cox & Snell R square 0.46; Hosmer & Lemeshow Goodness of fit *p* = 0.99

Reference category is MPO-ANCA

### Response to therapy

Forty-five patients (60 %) responded to the induction therapy in 4 [2; 6] months. Neither the histopathologic class nor the ANCA specificity influenced the rate of response.

In multivariate Cox regression analysis, although older patients and those with higher platelets number responded better, the need for dialysis at presentation was the most powerful predictor of response: the hazard ratio of response was two and a half times higher in patients not needing dialysis at presentation (Table 3).

**Table 3:** Predictors of the response to therapy; Multivariate Cox proportional hazards regression analysis

Variables	B	S.E.	Exp. (B)	95 % CI		Sig.*
Age >65 years (No)	−0.87	0.35	0.42	0.21	0.84	0.01
Ln (Platelets)	1.36	0.51	3.91	1.44	10.59	0.01
Albumin	0.78	0.40	2.18	0.99	4.77	0.05
Dialysis at presentation (No)	0.91	0.52	2.47	0.89	6.87	<0.0001

*B* coefficient b, *S.E.* standard error of coefficient b, *Exp. (B)* hazard ratio, *CI* confidence interval, *Ln* logarithm

*p ≤ 0.01 statistically significant

### Kidney outcome

Of the 51 patients alive at the end of the observation period, 38 (51 % of the whole cohort) were dialysis-free.

As compared to dialysis dependent-patients, those dialysis-free were older females and had less severe kidney disease at presentation (lower creatinine levels, lower proteinuria and hematuria). The kidney survival was not influenced by the vasculitis severity, ANCA serology or the histopathological class (Table 4).

**Table 4:** Patients’ characteristics according to kidney outcome

Parameter	End stage renal disease		*P* value
	No	Yes	
Patients number	38	13	
General			
Gender (% Male)	47	69	0.01
Age (years)	63 [53; 69]	51 [35; 57]	0.01
Vasculitis			
PR3-ANCA (%)	64	36	0.37
MPO-ANCA (%)	78	22	
Severe lung hemorrhage (%)	24	39	0.30
BVAS	15 [13; 21]	16 [15; 21]	0.63
Response to induction therapy	97 %	15 %	<0.0001
Inflammation			
Hemoglobin (g/dL)	9.0 [7.8; 10.0]	8.0 [6.0; 9.0]	0.07
WBC (per mm^3^)	9700 [7800; 14200]	8000 [6300; 12500]	0.17
ESR (mm/1 h)	99 [65; 115]	110 [82; 140]	0.28
Fibrinogen (mg/dL)	717 [578; 800]	720 [612; 820]	0.97
Albumin (g/dL)	3.8 [3.2; 4.0]	3.5 [3.1; 3.9]	0.25
Kidney			
Histopathologic class			
Crescentic (%)	46	67	0.28
Focal (%)	7	0	
Mixed (%)	33	29	
Sclerotic (%)	13	5	
Creatinine (mg/dL)	4.0 [2.9; 5.8]	9.0 [4.5; 12.0]	0.006
Proteinuria (g/day)	0.6 [0.4; 1.5]	2.3 [1.2; 3.0]	0.007
Hematuria (per mm^3^)	210 [150; 290]	460 [230; 980]	0.003
Dialysis at presentation (%)	8	62	<0.0001

Values are median [quartile 1; quartile 3] if not otherwise specified

*PR3-ANCA* patients positive for anti-proteinase 3 antibodies, *MPO-ANCA* patients positive for anti-myeloperoxidase antibodies, *BVAS* Birmingham Vasculitis Activity Score, *WBC* white blood cells, *ESR* erythrocyte sedimentation rate

**Fig. 2:**
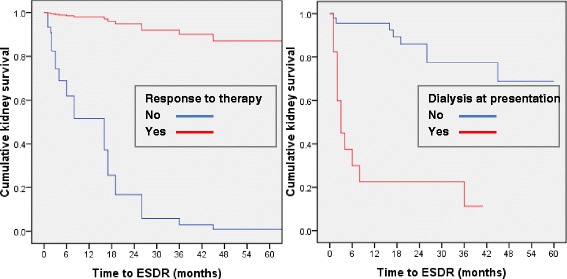
Adjusted cumulative kidney survival according to the response to induction therapy (left) and to the need of dialysis at presentation (right). Legend: The median ESRD-free survival time was longer in responders to induction therapy [4.5 (4.1–4.9) vs. 1.0 (0.3–1.6) years; *p* < 0.0001] and in those who did not need dialysis at presentation [4.1 (3.6–4.7) vs. 0.9 (0.3–1.6) years; *p* < 0.0001]. ESRD End stage renal disease

The unadjusted probability of kidney survival 1 and 5 years was 93 % and 64 %. The median ESRD-free survival time was longer in responders to induction therapy and in those who did not need dialysis at presentation (Fig. 2). In a Cox PHR model, the response to induction therapy and dialysis at presentation were retained as independent predictors of kidney survival. The responders had thirty four times more chances to avoid ESRD (HR = 33.88, 95 % CI 9.13–125.55, *p* < 0.001), while those not needing dialysis at presentation had a 84 % lower risk of kidney death (HR = 0.16, 95 % CI 0.05–0.48, *p* = 0.001).

### Patient outcome

Fifty one (68 %) patients survived. The main cause of death was pulmonary hemorrhage (*n* = 9; 38 % of deaths).

Surviving patients had less severe vasculitis (lower BVAS), less severe kidney disease (lower creatinine and hematuria, and fewer needed dialysis at presentation) and higher serum albumin levels. They responded in higher proportion to induction therapy. ANCA specificity and the histopathological class did not influence the patient outcome (Table 5).

**Table 5:** Patients’ characteristics according to outcome

Parameter	Patients’ death		*P* value
	No	Yes	
Patients number	51	24	
General			
Gender (% Male)	53	38	0.21
Age (years)	60 [50; 68]	63 [60; 68]	0.14
Vasculitis			
MPO-ANCA (%)	65	35	0.31
PR3-ANCA (%)	78	22	
Severe lung hemorrhage (%)	29	39	0.39
BVAS	15 [14; 21]	20 [17; 21]	0.01
Response to induction therapy	76 %	25 %	<0.0001
Inflammation			
Hemoglobin (g/dL)	8.6 [7.7; 9.9]	8.5 [7.4; 9.8]	0.79
WBC	9400 [7600; 13900]	10600 [7800; 18400]	0.21
ESR	100 [78; 120]	80 [61; 120]	0.20
Fibrinogen	720 [578; 820]	645 [525; 750]	0.15
Serum albumin	3.7 [3.2; 4.0]	3.2 [2.8; 3.6]	0.008
Kidney			
Histopathologic class			
Crescentic (%)	49	58	0.5
Focal (%)	4	8	
Mixed (%)	33	29	
Sclerotic (%)	14	4	
Serum creatinine (mg/dL)	4.7 [3.0; 7.4]	6.9 [4.1; 8.0]	0.06
Proteinuria	0.9 [0.5; 2.2]	0.8 [0.6; 2.2]	1.00
Hematuria	230 [180; 670]	365 [240; 1600]	0.008
Dialysis at presentation (%)	22	38	0.15

Values are median [quartile 1; quartile 3] if not otherwise specified

*PR3-ANCA* patients positive for anti-proteinase 3 antibodies, *MPO-ANCA* patients positive for anti-myeloperoxidase antibodies, *BVAS* Birmingham Vasculitis Activity Score, *WBC* white blood cells, *ESR* erythrocite sedimentation rate

**Fig. 3:**
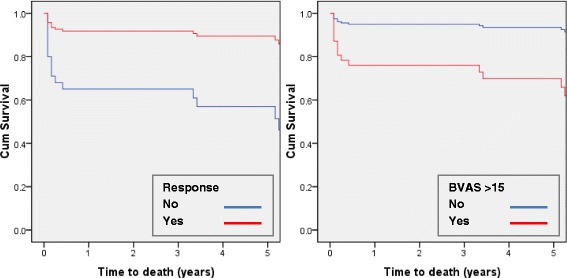
Adjusted cumulative patient survival according to the response to induction therapy (left) and to BVAS (right). Legend: The median survival was 8.4 (7.1–9.6) years in responders vs. 4.7 (2.9–6.8) years in non-responders, *p* < 0.0001 (left) and 7.5 (6.5–8.6) years in those with BVAS ≤ 15 vs. 6.1 (4.5–7.6) years in those with BVAS > 15, *p* < 0.0001 (right). BVAS Birmingham vasculitis activity score ver 3

The median survival was 7.2 years, and the cumulative survival 1 and 5 years was 88 % and 67 %. In a Cox PHR model, the independent predictors of death were a higher BVAS - for each increase in BVAS with three units, the risk of death was six times higher (HR = 5.88, 95 % CI 1.03–33.58, *p* = 0.05) - and the response to induction therapy - non-responders had five times less chances of survival (HR = 0.20, 95%CI 0.07–0.53, *p* = 0.0001) (Fig. 3).

## Discussion

This study reports the outcome in a cohort of Central East European patients with pauci-immune crescentic glomerulonephritis, where MPO-ANCA specificity was predominant, and the kidney damage severe and associated with lung hemorrhage. The patient and kidney outcomes were mainly influenced by the severity of kidney damage at presentation and by the response to induction therapy, but not by ANCA specificity, suggesting that the severity of organ lesions could overcome the ANCA-related phenotypic differences. Moreover, the severity of kidney lesions influenced the patients’ distribution in the histopathological classes, which probably limited its prognostic utility. In our cohort, median age was 60 years and gender distribution was balanced, as was reported in other series [1–3, 11, 18, 27–30]. The majority of cases were diagnosed in cold seasons, which suggest that upper respiratory tract infections were triggers of autoimmunity [31, 32]. The median lag between first symptoms and diagnosis was 2 months, highlighting the difficulty to recognize these protean diseases, in line with other reports [1, 29].

The main feature of this cohort was severe kidney disease (median creatinine 5 mg/dL; 27 % of patients needed dialysis at presentation). As in other series of vasculitis with severe kidney involvement [8, 30], the clinical condition was poor at presentation; 25 % of patients had BVAS >21. The lung was the second most affected organ; 31 % of patients had severe hemorrhagic alveolitis. Moreover, there was a strong correlation between the severity of kidney and lung disease. The odds of severe hemorrhagic alveolitis were four times higher in patients needing dialysis at presentation. Although a high frequency of lung involvement in patients with severe kidney disease was observed in other series [7], the clinical significance of this association had not yet been underlined. It could be explained by the severity of vasculitis involving both organs, amplified by the consequences of severe uremia on capillary permeability favoring lung bleeding. MPO specificity was 3 times more frequent than PR3 specificity, lower than reported in the United Kingdom and Norway, but closer to reports from Spain, which supports a South to North gradient of MPO specificity distribution across Europe [33]. In this cohort with MPO-ANCA dominance and severe kidney disease, male gender and the need for dialysis at presentation were significantly associated with PR3-ANCA when serum creatinine was above 5 mg/dL. This is in contrast with other studies which reported similar [1, 4, 34] or worse [9] kidney disease at presentation in MPO-ANCA, not in PR3-ANCA. This discrepancy could be explained by the faster pre-admission deterioration of kidney function in PR3-ANCA [4] or by a combination of late referral with a selection bias, as patients were specifically referred because of severe kidney damage. On the other hand, the severity of kidney disease at presentation could have offset the phenotypic differences between MPO and PR3. However, our data suggest that in regions where MPO-ANCA incidence is high, the possibility of severe kidney disease in PR3-ANCA patients in a nephrology setting should be kept in mind.

The rate of complete remission was 60 %, lower than that reported in controlled trials (75–85 %) [35–37], where responsiveness was related mainly to the therapeutic protocols, but closer to that reported in observational studies (50–75 %) [27, 30, 38, 39]. Possible explanations of the low rate of response are the severe clinical presentation and the infrequent use of plasmapheresis. In line with other cohorts of patients with severe kidney disease, in our patients the kidney failure described by the need for dialysis at presentation was an independent predictor of resistance to therapy while the ANCA pattern was not [8, 27, 38]. Thus, in a nephrology setting, the severity of kidney disease seems to overcome the prognostic significance of other factors. Interestingly, a higher platelets number was related to a positive response, as originally observed and interpreted by Westman [2] as reflecting a more severe immunological injury, not necessarily accompanied by more inflammation. This is also supported by our data showing higher albumin levels in responders and no difference in ESR or fibrinogen levels. Contrary to other series, older age was an indicator of a positive response. Possible explanations are a lower intensity of immune injury in elderly and increased disease awareness in elderly driving them to seek medical assistance in our particular population.

In spite of severe kidney disease at presentation, 51 % of our patients were alive and dialysis free at the end of the observation period, which is similar to reports from studies enrolling vasculitis patients with severe kidney dysfunction (56–64 %) [29, 40]. Moreover, the probability of kidney survival 5 years was comparable in our cohort to that reported in patients with similar kidney dysfunction at presentation [2, 29]. Unlike other studies where markers of kidney damage (serum creatinine [1, 2]), urinary IgM [41] or ANCA pattern [2, 28] predicted ESRD, in our series the resistance to induction therapy and the need of dialysis at presentation were the independent predictors of kidney death. On the other hand, 27 % of patients needing dialysis at presentation regained enough renal function to become dialysis-free at the end of the observation period. Accordingly, aggressive induction therapy is justified even in case of severe kidney failure. Simultaneously, it is to note that the severity of kidney status at presentation overwhelmed the severity of inflammation, the other organs lesions as well as ANCA specificity and the histopathological class as predictors of kidney death.

While kidney survival was associated with the initial organ damage, patient’s survival depended on vasculitis activity at presentation as reflected by BVAS. Several studies reported associations between BVAS and long term outcome [3, 42]. We observed a 6 times increase in risk of death for each three units rise in BVAS, which is in line with data reported in the largest multinational cohort of patients with ANCA-associated vasculitis where a small but significant effect of BVAS on survival was also found [3]. Thus, our data support the prognostic utility of BVAS and suggest a link between the initial organs damage and the long term prognosis, even when the clinical picture is dominated by the severity of kidney disease.

The other independent predictor of death was the response to induction therapy; the responders had five times more chances of survival than non-responders. As induction therapy was conducted according to current guidelines, including dose adjustment to renal function and age, this further underlines the limits of the existing therapeutic regimens.

Other studies reported differences in long term survival related to ANCA specificities [27, 28] while in our data neither kidney nor patient survival was related to ANCA serology. Nevertheless, in the previously mentioned EUVAS cohort, no ANCA-related dissimilarities in outcome were found and Flossmann et al. commented that previously reported differences in outcome could have been artifactual, caused by case-mix and underpowered adjustment for age and kidney damage [3]. Alternatively, when the consequences of vasculitis are severe - i.e. extensive kidney or other organs damage - they can conceal the initiating mechanism (ANCA specificity).

A histopathological classification of ANCA-associated glomerulonephritis was recently proposed by Berden et al. [19], aiming to improve prognosis of renal outcome and, eventually, to guide therapy. In our cohort, the proportions were similar in the case of the crescentic and sclerotic classes (52 vs 55 % and 11 vs 13 %), higher in the case of the mixed class (32 vs 16 %) and lower in the case of the focal class (5 vs 16 %) than reported by Berden et al. [19]. These differences could be due to a selection bias, as our patients were selected by the severity of kidney damage (which resulted in a lower frequency of focal class and a higher one in the mixed class), or to a limited reproducibility of the classification, as suggested by Ford et al. [20]. In our data, the histopathological classes were related neither to kidney function at presentation nor to kidney or patient outcome. However, our study had a low number of participants, which imposes caution in interpretation. Nevertheless, the validity of this classification was evaluated only in few studies so far and further research is clearly needed [20–23].

There are some limits of our study. The number of participants was low and the period of observation rather short, both limiting the statistical power. The single center design could be a limit for extrapolation of the results to other population, but data on Central East European population are scarce and on the other hand, this design increases the accuracy of data collection. ANCA were assessed by indirect immunofluorescence and/or by ELISA, and discrepancies between results obtained by these methods were described.

## Conclusions

In ANCA positive pauci-immune crescentic glomerulonephritis, the kidney and patient outcomes are related to the severity of kidney damage at presentation and to the response to therapy, but not to ANCA specificity or to the histopathological class. Thus, “the blast” - the acute injury inducing severe organ damage - results in a peculiar distribution in histopathological classes, reduced responsiveness to therapy and poorer outcome, and could hide the prognostic influence of both “the trigger” - i.e. ANCA specificity - and of the histopathology lesions.

## Abbreviations

MPO: Myeloperoxidase
PR3: Proteinase 3
ANCA: Anti-neutrophil cytoplasmic antibodies
BVAS: Birmingham Vasculitis Activity Score ver. 3
ESRD: End stage renal disease
ELISA: Enzyme-linked immunosorbent assay
CI: Confidence interval
PHR: Proportional hazard ratios
HR: Hazard ratio
EUVAS: European vasculitis study group
CNS: Central nervous system
WBC: White blood cells
ESR: Erythrocyte sedimentation rate
